# Approaches to clinical guideline development in healthcare: a scoping review and document analysis

**DOI:** 10.1186/s12913-022-08975-3

**Published:** 2023-01-16

**Authors:** Annemarie De Leo, Dianne Bloxsome, Sara Bayes

**Affiliations:** 1 Edith Cowan University, 270 Joondalup Drive, Perth, WA Australia; 2 Australian Catholic University, 8-14 Brunswick St. Fitzroy, Victoria, Australia

**Keywords:** Clinical Practice Guidelines, Guideline development, Evidence-based medicine, Standardisation, Quality healthcare

## Abstract

**Background:**

Over the past decade, an industry has emerged around Clinical Practice Guideline (CPG) development in healthcare, which has increased pressure on guideline-producing organisations to develop CPGs at an accelerated rate. These are intended to improve the quality of care provided to patients while containing healthcare costs and reducing variability in clinical practice. However, this has inadvertently led to discrepancies in CPG recommendations between health organisations, also challenging healthcare providers who rely on these for decision-making and to inform clinical care. From a global perspective, although some countries have initiated national protocols regarding developing, appraising and implementing high-quality CPGs, there remains no standardised approach to any aspect of CPG production.

**Methods:**

A scoping review of the literature and document analysis were conducted according to Joanna Brigg’s Institute methodology for scoping reviews. This comprised two qualitative methods: a comprehensive review of the literature (using CINAHL, Scopus and PubMeD) and a document analysis of all national and international guideline development processes (manual search of health-related websites, national/international organisational health policies and documents).

**Results:**

A set of clear principles and processes were identified as crucial to CPG development, informing the planning, implementation and dissemination of recommendations. Fundamentally, two common goals were reported: to improve the quality and consistency of clinical practice (patient care) and to reduce the duplication or ratification of low-grade CPGs.

**Conclusions:**

Consultation and communication between CPG working parties, including a wide range of representatives (including professional organisations, regional and local offices, and relevant national bodies) is essential. Further research is required to establish the feasibility of standardising the approach and disseminating the recommendations.

## Introduction

In the last 20 years, the number of Clinical Practice Guidelines (CPGs) produced for healthcare has risen exponentially [1]. CPGs are perceived to present best evidence for managing clinical matters, including conditions or symptoms, and are upheld as the gold standard of high-quality healthcare [2]. They offer a way of bridging the gap between what is known to be best evidence, policy and gold practice standards in healthcare [3, 4]. Produced by various local, national and international organisations, CPGs have traditionally been defined as a set of ‘systematically developed statements aimed at helping people make clinical, policy-related and system-level decisions’ [5]. A more contemporary proposition is that guidelines offer a mechanism for packaging evidence and presenting recommendations to healthcare decision-makers [1]. CPGs have a range of common purposes: they include statements that establish best practice standards, provide benchmarks for clinical audits, strive toward improving the quality of healthcare delivery at an organisational level, and provide guidance on particular clinical practices [6]. Yet, there is inconsistency in the principles underpinning CPG development and the processes leading to best practice recommendations.

Over the past decade, an industry around CPG development has increased efforts by guideline-producing organisations to develop CPGs at an escalated rate [4, 7]. To facilitate this process, several collegiate groups have each presented an approach to clinical guideline development in the form of guideline development manuals [8–11]. There are possibly many more health organisations, local departments and professional associations that have produced recommendations for developing clinical care or standardised practices, each of which may have adopted its own approach to identify, appraise, synthesise and describe the evidence-based underpinning best practice recommendations [6]. To the best of our knowledge, however, there remains no standardised approach to any aspect of CPG production.

### Problems and new approaches: mapping the way forward

Various problems with guideline development processes have been reported in the past, which impede their optimal use and impact at the point of care [12]. In 2003, Grol identified a ‘guideline industry emerging in many western countries’ (p. 55), reporting considerable variation in recommendations, their quality and application to clinical practice at that time [3]. This was thought to result from ad hoc approaches to CPG development processes and recommendations not based on the best available evidence. Brouwers and Kho [5] identified poor coordination between national and local level guideline developers to be another contributing factor, leading to unnecessary duplication of low-quality CPGs, inconsistency in recommendations for best practice and sub-optimal care for patients.

Since then, approaches to CPG development have made significant strides in refining and describing the requirements for high quality CPGs [13, 14], although these advancements have not matched the publication rate of the latest scientific literature or the emerging practice issues that clinicians and policymakers are challenged by [15, 16]. This bears out concerns raised by Grol (2003), who highlights various issues with existing guidelines (i.e. lack of quality and consistency) and the translation of latest evidence into best practice recommendations [3]. Additionally, Louw et al. [2] were apprehensive towards stakeholder involvement in CPG production, suggesting they have varied experience of the process or knowledge of clinical matters; in Joyce and Cartwright’s [17] view, this contributes to the production of CPGs, which at times fail to meet international quality criteria or the needs of clinicians working in practice environments.

In an effort to ensure CPGs are robust and reliable as intended, a range of ‘next stage’ approaches to CPG development have emerged in recent years, all of which focus on optimising methodological transparency [18, 19]. While these offer a degree of standardization, there remains inconsistency in their approach to CPG development. One example is the collaboration between Cochrane South Africa, the South African Medical Research Council (SAMRC), the Centre for Evidence-based Health Care (CEBHC) and the International Centre for Allied Health Evidence (iCAHE), who together produced an online CPG-development Toolkit to assist individuals who are interested in knowing how to develop context-specific CPGs [20]. An alternative approach, the ADAPTE Collaboration, is an international partnership between researchers, guideline developers and implementers who promote the adaptation of existing guidelines, developing a manual and resource that outlines a process for upgrading CPGs produced in one setting for use in other contexts [21]. From a global perspective, national and international health organisations increasingly issue their own CPGs, which has caused various discrepancies, duplication and sometimes contradictory recommendations between healthcare sites and recommendations for clinical care [16, 18, 22]. Although some countries have initiated national protocols regarding the development, appraisal and implementation of CPGs, many are yet to establish a standardised approach [23]. Louw et al. [2] suggest transparency in CPG development processes is another crucial consideration for improving the quality and consistency of clinical care, both locally and globally.

Evidence suggests that increased collaboration between local, organisational and regional CPG working parties may improve the quality of health services on a global scale [24]. Similarly, communication and coordination among interdisciplinary CPG developers may reduce the duplication and variability of best practice recommendations between health organisations [23]. Collaborations such as the Guidelines International Network (GIN) [25] and the Institute of Medicine (IOM) [26] have each established a standardised approach to clinical guideline development, aiming to streamline the production and dissemination of regional guidelines. Additionally, global organisations such as the World Health Organisation (WHO) and the Swiss Centre for International Health (SCIH) have developed guiding principles to strengthen health systems, suggesting an approach that advocates for interdisciplinary and multisectoral collaboration would cater to different contexts and countries around the world.

This review aimed to explore evidence underpinning the processes and principles of health-related CPG development, including handbooks and methodological guidance publications. Although evidence exists on specific health organisations’ approach to CPG development, exploration of their principles and processes may inform the development of a standardised approach that is acceptable to healthcare providers and health organisations worldwide, and CPGs that present best practice recommendations based on the latest evidence.

### Aim

This review aimed to elicit information on what is known about clinical practice guideline development in healthcare. Our review question was: *“What is known about approaches to clinical guideline development in healthcare?* To achieve this aim, two specific objectives were identified:

• Establish the various principles applied to clinical guideline development; and
• Determine the processes by which this occurs.

## Methods

To address the objectives above, we employed two complementary qualitative research methods: the first comprised a scoping review of the literature, and the second included document analysis of all national and international guideline processes regarding CPG development. Although different, both methods are considered interrelated qualitative approaches for conducting thematic data analysis and interpretation [27].

### Study design

Two methodological approaches guided the scoping review. First, the Joanna Briggs Institute (JBI) methodology for conducting scoping reviews [28], which provides the most current method for scoping reviews and draws on the approach of Askey and O’Malley [29]. The steps involved: formulation of the research question, identification and retrieval of relevant studies, quality appraisal of the selected studies, data extraction through coding, synthesis and reporting of finding [30]. Second, document analysis was performed on policy and government records relevant to CPG development. This complimentary qualitative method entailed finding, selecting, appraising and synthesising data to create meaningful categories and themes by following a systematic process. The choice to include document analysis in this review rests on the fact that obtaining convergence through the use of different data sources strengthens the impact and credibility of the findings, also referred to as triangulation [31].

### Data collection

Original articles, reviews and health-related CPG documents from inter-governmental and non-governmental organisations were included if they met the inclusion criteria.

### eligibility criteria and document selection

#### Population

The review was not limited to a specific healthcare population. All health organisations and disciplines within healthcare were included in this review.

### Concept

Given the review was designed to elicit information about intercollegiate guideline networks and other approaches relevant to clinical guideline development, we considered documents that provided a definition or description of CPG development relevant to the health industry.

### Context

We considered all literature relevant to clinical guideline development.

### Type of documents

We considered all open-access literature published between 2000–2022. Health-related policy and government documents, reviews and primary research articles written in English were considered for inclusion. Additional literature and health-related CPG documents were also sought from health-related organisational websites.

**Fig. 1:**
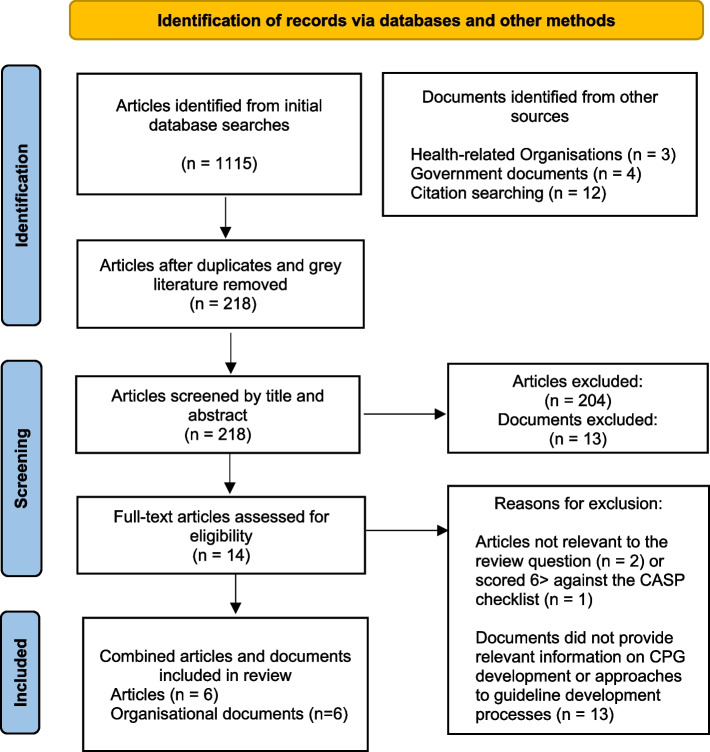
PRISMA flow diagram

We attempted to identify records that defined or discussed approaches to health-related CPG development. Following a cursory search, date parameters were set between 2000–2022, as seminal work on guideline development was noted during this timeframe. Records were included if they identified key stakeholders of health-related clinical guideline development networks, mapped CPG processes or discussed key principles of CPG development. Documents were excluded if they were not published in English or relevant to the review question and objectives. Following screening, available full texts were retrieved, reviewed and tabulated by author one (see Fig. 1).

### Search strategy

In accordance with the JBI approach, we employed a three-step search strategy. First, a preliminary search was conducted in Cumulative Index to Nursing and Allied Health Literature (CINAHL), a broad database that indexes high-quality literature relevant to nursing and allied health, health research, healthcare and health education. The search terms used were health* AND (“guideline development” OR “intercollegiate network” OR “international network” OR “clinical guidelines process”) AND (“care maps” OR “clinical guidelines” OR “practice guidelines”). This preliminary search was followed by an analysis of the keywords in the title and abstract of retrieved documents and the index terms used to describe the documents. We identified the following search terms, which were added to the initial search terms applied: “clinical care process specifications”, “caremaps” and “practice guidelines”. Suitable MeSH or Subject headings were not identified.

Second, we conducted a database search using all identified keywords in CINAHL, Scopus and PubMed. Third, a manual search through the reference lists of all identified documents was conducted for additional relevant documents. The first author also researched health-related websites for policy or government documents relating to CPG development. This was conducted by entering various combinations of the original search terms in Google, followed by a manual search for references to CPGs in the articles retrieved during the initial search. The following CPG developers were identified: “Health and Medical Research Council of Australia (NHMRC)”, The Joanna Briggs Institute (JBI)”, “American Agency for Healthcare Research and Quality (AHRQ)”, “Guidelines International Network (GIN)”, UK National Institute of Health and Care Excellence (NICE)” and the “Scottish Intercollegiate Guidelines Network (SIGN)”.

### Quality appraisal

Given the diversity and multidisciplinary nature of the data, quality appraisal was performed initially by categorising the sources of data into two different groups. The first group comprised peer-reviewed articles, the second group included data sourced from all other documents (web-based content and health-related organisational guideline development documents). We considered the sources in the first group to be of higher quality, given that the documents were subjected to peer review. This was performed by author one, who assessed each article's methodological quality for inclusion against the JBI CASP checklist. Articles that scored > 6 out of ten were deemed high quality and included in the review.

### Data extraction, coding and analysis

Data extraction was undertaken in three stages by the first author. First, key information from each text was obtained. This included author(s) names, publication date, country, record type, aim(s) and key concepts or principles presented in the results. Second, thematic analysis was conducted on the first data set (which comprised peer-reviewed articles) following Braun and Clarke’s six-stage guide to thematic analysis [32]. This was conducted iteratively; data were coded, categorised and reviewed independently by each author. Following this step, the authors independently reviewed each category and exchanged ideas with each other until a final agreement was made on the resulting categories. Third, document analysis was conducted on the second group of data (comprising organisational documents), which is often used when authors seek convergence through different data sources and methods [31]. This comprised reading each document, coding information that was relevant to the review question and objectives, analysing the findings and comparing these with the data extracted from the articles included in this review. Similar to thematic analysis, document analysis is the process of organising information into meaningful codes that inform the central research question [31]. The summarised findings were presented as core categories underpinned by the sub-categories and initial findings.

## Results

Six articles were included in this review, and five health-related organisational documents, which collectively presented current information on various approaches to CPG development in healthcare. Of these, perspectives and approaches were included from Australia, Canada, the United Kingdom (UK), Asia, South Africa, Scotland and the United States of America (USA). All articles discussed, to some extent, processes by which guideline development groups function, collaborate and work through the guideline development process. Similarly, all documents explained the processes and methods used in CPG development (see Table 1). The findings presented a set of common principles and processes that could guide future discussions about CPG development processes.

**Table 1:** Document and article characteristics

Record	Country	Record type and reference number	Record aims	Key concepts/principles/processes/themes
Boltin, Lambregts, Jones, Siterman, Bonovas, Cornberg, Khannoussi, Doherty (2020)	Asia	Review article (Ref [18])	To establish the steps necessary to initiate guideline development processes	Key themes presented: • Identifying the unmet need • Defining the scope • Building working groups • Managing conflicts of interest • How to search and appraise the evidence • Guideline implementation • Writing guidelines
Eccles, Grimshaw, Shekelle, Schunemann, Woolfe (2012)	United States of America	Methodology article (Ref [33])	To determine the processes by which guideline development groups function and the important procedural issue of managing conflicts of interest during guideline development	Key findings: • Prioritising topics for guideline development • Consumer involvement • Guidelines group processes • Managing conflicts of interest
Garbi (2021)	United Kingdom	Review article (Ref [34])	To describe the NICE clinical guidelines development principles and processes To provide an informed perspective on the recommendations made	Key principles identified: • Guideline committees should include a wide representation of stakeholders • Scoping the guideline • Sourcing clinical and economic evidence to support CPG development • The power of consultation • Publication and implementation
Hill, Bullock, Alderson (2011)	United Kingdom	Original article (Ref [35])	To establish the processes used to produce CPGs based on best available evidence	Key principles of the guideline development process: • Selection of the focused topic • Recruitment of guidelines development group • Guideline development • Consultation • Preparation for implementation • Validation and implementation
Kredo, Bernhardsson, Machingaidze, Young, Louw, Ochodo and Karen (2016)	South Africa	Research article (Ref [23])	To provide a guide that describes the standards, methods and systems reported in current CPG methodologies and implementation literature	• CPG activities are evolving processes • The standards, methods and systems in use by those involved with CPGs provide evidence of international guideline activity • There is a need to build on the knowledge of current activities in CPG development to optimise end-user engagement and impact global health outcomes
National Health and Medical Research Council (NHMRC) (2009)	Australia	Document (Ref [10])	This document puts forward a method for developing such guidelines in Australia	Key principles presented: • Focus on outcomes • Guidelines should be based on the best available evidence (taking the best available evidence and turning it into a clinically useful recommendation) • CPG development depends on the judgment, experience and good sense of the working party • The process of guideline development should be multidisciplinary • Guidelines should be flexible and adaptable to varying local conditions (also including evidence relevant to different target populations, geographic and clinical settings, and make provision for the different values and preferences of patients • Guidelines should be developed with resource constraints in mind • Consideration for end-users is crucial
National Institute for Health and Care Excellence (NICE) (last updated 2020)	United Kingdom	Document (Ref [9])	To provide an explanation for the processes and methods NICE uses for developing, maintaining and updating NICE guidelines	Key principles outlined: • Guidelines are based on the best available evidence of what works, and what it costs • Guidelines are developed by independent and unbiased committees of experts • Guidelines committees should include at least 2 lay members (people with personal experience of using health or care services, including carers, or from a community affected by the guideline) • Regular consultation allows organisations and individuals to comment on final recommendations • Once published, all guidelines should be regularly checked and updated in light of new evidence or intelligence • Guidelines committee should ensure that processes, methods and policies remain up-to-date
Quaseem, Forland, Macbeth, Ollenschlager, Phillips, Van de Wees (2012)	United Kingdom	Document (Ref [36])	To promote discussion and consensus on a set of international standards for guideline development	Considerations for high-quality guideline development: • Composition of the guideline development group • Decision-making process • Conflicts of interest • Scope of the guideline • Peer review and stakeholder consultation
Schünemann, H., Brożek, J., Guyatt, G., & Oxman, A (2013)	Canada	Document (Ref [4])	To develop a common, sensible approach to grading the quality of evidence and strength of recommendations based on the approach proposed by the Grading of Recommendations, Assessment, Development and Evaluation (GRADE) Working Group	Key processes highlighted: • Processes are interrelated and not necessarily sequential. The panel works collaboratively, informed through consumer and stakeholder involvement • Considerations for the organization, planning and training encompass the entire guideline development process, and steps such as documenting the methodology used and decisions made, as well as considering conflict-of-interest occur throughout
Scottish Intercollegiate Guidelines Network (SIGN) (2015)	Edinburgh, Scotland	Document (Ref [11])	To provide a reference tool that may be used by guideline development groups to improve the quality of health care for patients by reducing variation in practice and outcome	Key principles: • Composition of the guideline development group • Selection of guideline topics • Conducting a systematic literature review • Assessing the quality of evidence • Making recommendations • Consultation and peer review • Presentation and publication • Implementation • Involving consumers and their representatives
The World Health Organization (2012)	Switzerland	Document (Ref [8])	To provide a clear map for the process of guideline development, that guidelines have credibility and meet WHO’s criteria for content, methods and presentation	Key concepts presented: • Defining the guideline scope • The importance of clear guideline processes • Defining key stakeholders and working with external partners • The value of peer review • Dissemination and evaluation of clinical guidelines

### Findings from the literature

#### The working party: composition and structure

The most consistent approach to CPG development appears to come from the formulation of a working party, which, although referred to using different terminologies (for example a guideline panel, guideline committee, guideline development group and steering committee), was consistently reported to include individuals from professional, organisational, regional and national levels [1, 37]. International consensus suggests that CPG working parties should be multidisciplinary and have a range of diverse and relevant stakeholders [33, 36]. This may consist of healthcare professionals who are directly involved in clinical care or management of patients, organisations that represent healthcare professionals, providers and commissioners of health services, manufacturers of medicines or healthcare equipment, policymakers who make decisions about resource utilisation, methodologists, topic experts and consumer representatives [34, 35]. Group members are selected for their pre-eminence to contribute to the working group process and attributes as effective team members [18]. Notably, groups that fail to form a multidisciplinary working party have been associated with clinical guideline recommendations that do not reflect evidence-based practice [36].

#### Guideline development processes and decision-making

Clinical guideline development was reported across all articles to involve both a technical process (searching and appraising evidence-based research) and a social process (translating evidence-based research into CPGs) [9, 10, 25, 37]. The outcome of both methods was also noted to be dependent upon the composition of the working group and whether the right people have been equally represented and involved throughout the process [33]. Similarly, stakeholders external to the core working party were considered an essential component of guideline development processes, with consumer representatives, external sponsors and members of the public highlighted as beneficial [35, 36]. Boltin et al. [18] went further to suggest that this was not only to provide peer review but to offer a ‘wide scientific, geographical and philosophical reach’ (p.855).

Specific guideline development processes were commonly reported as a series of steps or phases that mapped the pathway from CPG development to dissemination. This included: identifying the need for and scope of the CPG, recruitment of an interdisciplinary working group and engaging with key stakeholders, searching for evidence, developing best practice recommendations, external review and consultation, dissemination and implementation of recommendations [1, 18, 34, 35]. Ideal conditions for optimising this process were defined as those that enabled the views of all parties to be expressed and considered before a recommendation for practice was reached [36]. Notably, the optimal size for guideline development groups ranged from 10–20 persons, with larger working parties reported as being more challenging to manage. Comparatively, smaller groups lacked a diversity of relevant stakeholders [18, 34].

Group decision-making was generally reported as a formal process for reaching group consensus [36], involving three core phases: orientation (identifying the problem), evaluation (discussion of decision alternatives), and control (deciding which alternative is the best-fit option) [33]. However, some organisations also used other informal methods (such as relying on clinician perspectives and patient preferences) to make critical decisions or recommendations regarding clinical practice [1].

#### Managing conflicts of interest

An aspect consistently reported across all articles was the need to consider conflicts of interest (COI), given that financial, intellectual and other investments in all areas of healthcare could lead to biased judgement regarding the scope or topic of focus. Conflicts of interest were also noted to arise during the guideline development process, potentially introducing substantial bias in the final recommendation [18]. Similarly, COIs could misinform healthcare decision-makers, damaging working parties’ reputations or resulting in drawn-out processes for dealing with perceived COIs [33].

### Findings from document analysis

One national and five international health-related documents were examined to extract definitions and other relevant information regarding approaches to CPG development [8–11, 36]. Based on the analysis of these documents, it was possible to compare their approaches; and explore the various principles and processes between them.

There was international consensus that guideline development groups should be multidisciplinary, gender and geographically balanced, representing all those likely to use the intended clinical guideline (both professional and consumer) [8, 11, 36]. This view also extended to include national and international collaborations, persons from rural and urban locations and specialists other than clinicians (i.e., Health economists and social workers) [11, 36]. In addition to these attributes, the primary aim of the working group was defined as needing to be outcome focused [9–11].

#### Principles of CPG development

CPG development was described by two organisations as a set of critical principles that presented the best available evidence with resource constraints in mind, taking into account the anticipated end users or groups most likely to be affected by the recommendations [8, 11]. Similarly, guideline development was described as the method used to develop, maintain and update CPGs [9].

Each of the six documents included in this review individually outlined a set of core principles considered essential for developing CPGs. When compared, the attributes underpinning good CPG development were identified, and the following summations were made:

• Guidelines should be outcomes focused and involve a cycle of interdependent activities: Planning and development, dissemination, implementation and evaluation.
• Guidelines should be flexible and capable of adapting to varying local and global audiences.
• Guidelines should be based on the best available evidence and include a statement about the strength of recommendations.
• Guidelines should demonstrate essential qualities such as validity, reliability, clinical applicability, flexibility and clarity.
• Guidelines should be continually revised to maintain currency and update in light of new evidence or intelligence.
• Collaboration between local and national agencies, inter-governmental organisations and relevant expert opinion (both professional and consumer-led) is preferential.

**Fig. 2:**
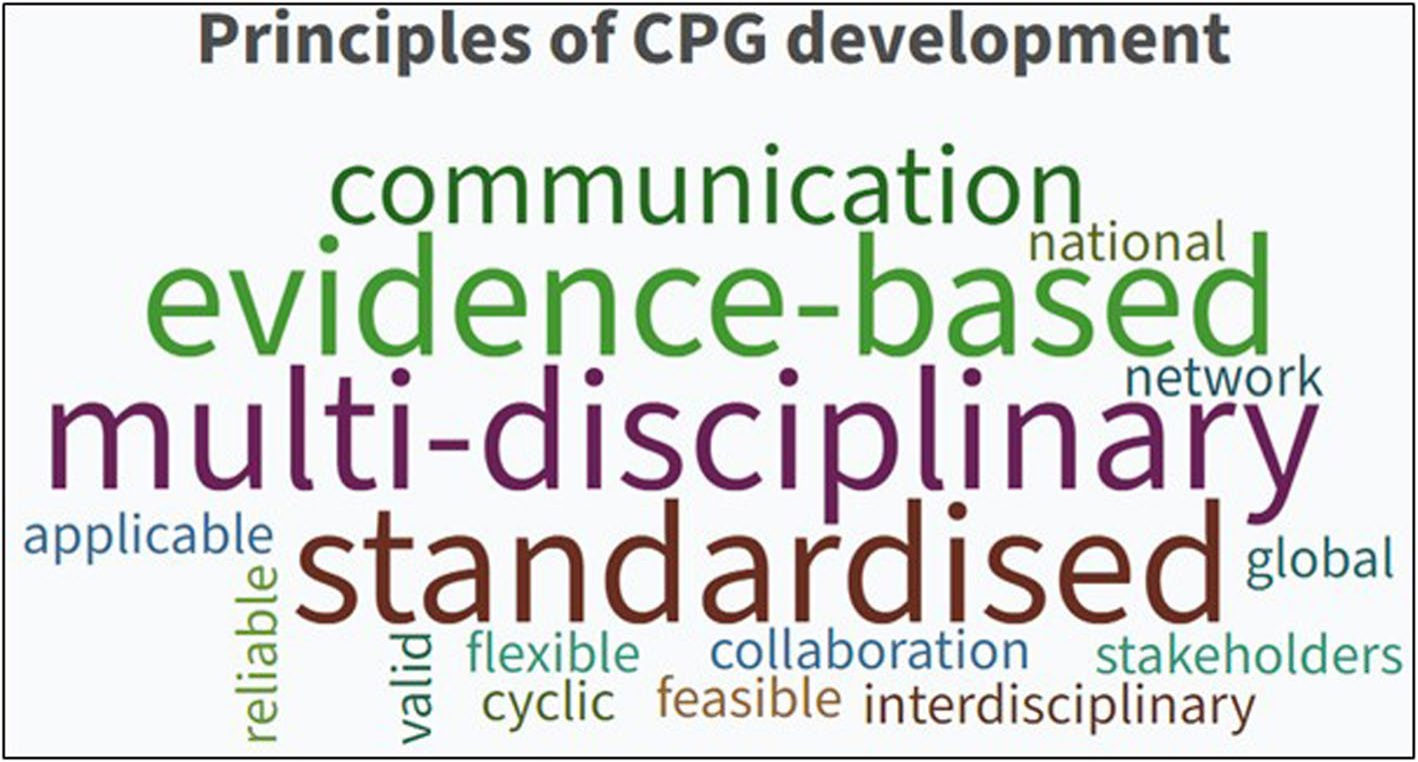
The most common words used to describe CPG principles

Combined word frequencies in all documents indicated that good principles of CPG development primarily relied on multidisciplinary collaboration, communication and a standardised approach (see Fig. 2).

#### Processes for CPG development

All documents, to some degree, referred to CPG development as a process of identifying and implementing interventions (including practices) to optimise the best possible health outcomes for consumers [8–11]. This also included the ideal group membership number, ranging from 10–20 members [8] to 12–18 members [9]. Additionally, all documents concurred that developing recommendations for clinical practice required a clear, comprehensive process based on all available evidence. The overarching concepts identified were collaboration (both inter-disciplinary and organisational), transparency regarding the approach and ongoing revision to the guideline development process.

Formulation of a set of key processes for undertaking CPG development activities was established using iterative comparison and evaluation, which resulted in eight core processes consistently reported as essential to CPG development:

• Planning and defining the scope of the guideline.
• Formation of an inter-disciplinary, and where possible inter-organisational, guideline development panel.
• Defining the purpose of the guideline and intended target audience.
• Reviewing the literature and developing recommendations for practice.
• Stakeholder consultation (both internal and external) and peer review.
• Presentation and publication of the CPG.
• Dissemination and implementation.
• Evaluation and ongoing revision.

**Fig. 3:**
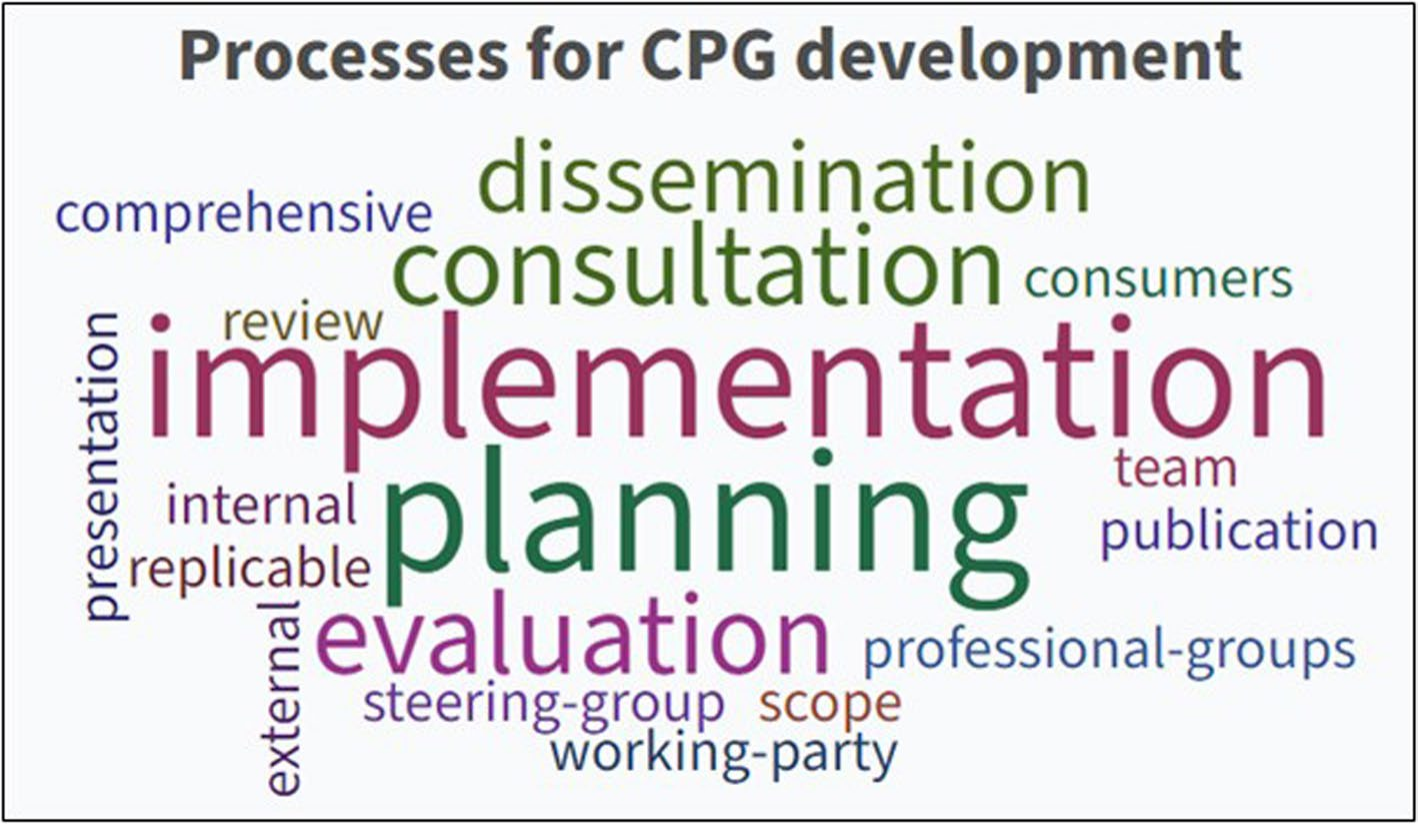
The most common words used to describe CPG processes

The thematic analysis results identified five common processes for CPG development: Planning, consultation, implementation, evaluation and dissemination (see Fig. 3).

To date, there has been no exploration or evaluation of the varying approaches to CPG development worldwide. Yet, clinicians, consumers and healthcare organisations rely on these to guide clinical practice. The findings of this review identify the core principles and processes that can be used when developing CPGs, including the underpinning ethical and value-based activities that should guide the decisions of national and international guideline committees.

## Discussion

This review intended to present a clear overview of what is known to date about various approaches to CPG development in healthcare and the implications of this on health services, care providers and clinical outcomes. As a result, a set of clear principles and processes were identified as crucial to guideline development activities, which inform the planning, dissemination and implementation of CPGs. Fundamentally, all documents included in this review articulated two common goals: to improve the quality and consistency of clinical practice (patient care) and to reduce the duplication or ratification of low-grade CPGs. Unequivocally, clinicians want to provide patients with evidence-informed care. To achieve this, they require guidelines that reflect the evolving body of scientific evidence in combination with clinical expertise and patient preferences. This parallels evidence-based practice (EBP). Yet, in many areas across the health sector, knowledge translation and inconsistency in both policy and practice continues to hamper the closure of the evidence-practice gap in healthcare [16, 38]. To improve clinical practice standards and consumers' health outcomes, well-developed CPGs and effective processes for evidence implementation are needed [39]. The authors of this review found no comparable literature on this subject; however, acknowledge the purpose of this review was to collate and interpret what is published to date.

Globally, a surge in publications around CPG development indicates the increasing interest and research focus on facilitating EBP. It also confirms a rise in the number of CPGs developed for local, regional and system-level use [40]. These are intended to improve patients’ quality of care while reducing healthcare costs and variability in practice [41]. Several organisations responsible for producing evidence-based CPGs have published handbooks at a national level [9–11, 26, 42], seeking to minimise variations in clinical practice and standardise healthcare interventions at a national level. However, progress in developing such national guidelines, particularly in low and middle-income countries, remains relatively low [41]. Arguably, if CPGs were standardised through a national or international network, care providers and patients would benefit exponentially.

An international team of guideline developers and researchers, known as the AGREE collaboration (Appraisal of Guidelines, Research and Evaluation), sought to address this issue by creating a generic instrument, initially labelled the AGREE and then later amended to the AGREE II, which was designed to assess the rigour of guideline development processes [5]. However, the items and domains within this instrument focus mainly on methodological issues and do not guarantee optimal recommendations or better health outcomes for patients. This leaves health services and government departments without assured guidelines to inform local, regional and national standards of care.

At the core of this review, the requirement for consultation and communication between parties and collaboration from a wide range of representatives (including professional organisations, regional and local offices, and relevant national bodies) were highlighted as essential. These concepts resonate with other well-established national and global guideline development working parties [8, 42, 43], who concur that CPG development groups should reflect an interdisciplinary network that comprises users, consumers and expert representatives from both local and international contexts. Overarchingly, the findings of this review confirmed CPG recommendations should reflect the diversity of all representatives involved, focusing on supporting healthcare providers, health organisations and government bodies with evidence-based guidelines that are current, practical and easily transferrable.

This review has some limitations. There are possibly other guideline development organisations (for example, in Asia and Latin America) that may not have published principles or processes for CPG development yet provide clear guidance on these aspects for end users. As such, they were not identified during the search and screening process. There may also be other published literature to support the findings of this review that were not sourced. However, the broad inclusion criteria for this scoping review ensured all records (both published and web-based) were considered for inclusion and were not limited to document type.

## Conclusion

Our review aimed to elicit information on what is known about CPG development in healthcare. From the records included in this review, there is strong concordance as to the key principles and processes of CPG development: Establish a multidisciplinary guideline development group, have a wide range of experts from both local and regional contexts, identify the problem and develop recommendations that are applicable and transferrable across sites and health systems, collaborate and consult with persons both in and external to the guideline development group. While these key principles and processes are both useful to health service providers and decision-makers in healthcare contexts, there remains ongoing inconsistency in clinical practice and quality of care between health organisations around the world, excessive duplication of low-grade CPGs also wastes resources and the efforts of care providers who rely on CPGs to inform their decision-making and clinical practice. To address this persistent issue, further research is required to establish the feasibility of standardising the approach and resultant recommendations made to CPGs.

## Data Availability

All data and materials are available on request to author one (ADL).

## References

[1] Kredo T, Bernhardsson S, Machingaidze S, Young T, Louw Q, Ochodo E (2016). Guide to clinical practice guidelines: the current state of play. Int J Qual Health Care.

[2] Louw Q, Dizon JM, Grimmer K, McCaul M, Kredo T, Young T (2017). Building capacity for development and implementation of clinical practice guidelines. S Afr Med J.

[3] Grol R, Cluzeau FA, Burgers JS (2003). Clinical practice guidelines: towards better quality guidelines and increased international collaboration. Br J Cancer.

[4] Schunemann HJ, Wiercioch W, Etxeandia I, Falavigna M, Santesso N, Mustafa R (2014). Guidelines 2.0: systematic development of a comprehensive checklist for a successful guideline enterprise. CMAJ.

[5] Brouwers MC, Kho ME, Browman GP, Burgers JS, Cluzeau F, Feder G (2010). AGREE II: advancing guideline development, reporting and evaluation in health care. CMAJ.

[6] Zhang Y, Coello PA, Brozek J, Wiercioch W, Etxeandia-Ikobaltzeta I, Akl EA (2017). Using patient values and preferences to inform the importance of health outcomes in practice guideline development following the GRADE approach. Health Qual Life Outcomes.

[7] Umscheid CA, Agarwal RK, Brennan PJ (2010). Healthcare Infection Control Practices Advisory C Updating the guideline development methodology of the Healthcare Infection Control Practices Advisory Committee (HICPAC). Am J Infect Control.

[8] World Health Organisation. WHO Handbook for Guideline Development. 2014.

[9] National Institute of Health Care Excellence. Developing NICE guidelines: The manual. 2020. 26677490

[10] National Health and Medical Research Council. A guide to the development, implementation and evaluation of clinical practice guidelines. 2009.

[11] Scottish Intercollegiate Guidelines Network. SIGN 50: A guideline developer's handbook. 2011.

[12] Ollenschlager G (2004). Improving the quality of health care: using international collaboration to inform guideline programmes by founding the Guidelines International Network (G-I-N). Qual Saf Health Care.

[13] Rosenfeld RM, Shiffman RN, Robertson P (2013). Department of Otolaryngology State University of New York D Clinical Practice Guideline Development Manual, Third Edition: a quality-driven approach for translating evidence into action. Otolaryngol Head Neck Surg.

[14] Dizon JM, Machingaidze S, Grimmer K (2016). To adopt, to adapt, or to contextualise? The big question in clinical practice guideline development. BMC Res Notes.

[15] Toohill J, Sidebothom M, Gamble J, Fennwick J, Creedy D (2017). Factors influencing midwves’ use of an evidence based normal birth guideline. Women and Birth.

[16] Bayes S, Juggins E, Whitehead L, De Leo A (2019). Australian midwives’ experiences of implementing practice change. Midwifery.

[17] Joyce KE, Cartwright N (2020). Bridging the Gap Between Research and Practice: Predicting What Will Work Locally. Am Educ Res J.

[18] Boltin D, Lambregts DM, Jones F, Siterman M, Bonovas S, Cornberg M (2020). UEG framework for the development of high-quality clinical guidelines. United European Gastroenterol J.

[19] Smith LS, Wilkins N. Mind the Gap: Approaches to Addressing the Research-to-Practice, Practice-to-Research Chasm. J Public Health Manag Pract. 2018;24 (Suppl 1 Injury and Violence Prevention):S6-S11. 10.1097/PHH.0000000000000667. 10.1097/PHH.0000000000000667 PMC6051530 29189499

[20] SAGE Project. Guideline Toolkit 2021 [Available from: https://guidelinetoolkit.org.za/.

[21] Fervers B, Burgers JS, Voellinger R, Brouwers M, Browman GP, Graham ID (2011). Guideline adaptation: an approach to enhance efficiency in guideline development and improve utilisation. BMJ Qual Saf.

[22] De Leo A, Bayes S, Geraghty S, Butt J. Midwives' use of best available evidence in practice: An integrative review. J Clin Nurs. 2019;28(23-24):4225-35. 10.1111/jocn.15027 PMC7328778 31410929

[23] Kredo T, Cooper S, Abrams A, Daniels K, Volmink J, Atkins S (2018). National stakeholders' perceptions of the processes that inform the development of national clinical practice guidelines for primary healthcare in South Africa. Health Res Policy Syst.

[24] Alonso-Coello P, Irfan A, Sola I, Gich I, Delgado-Noguera M, Rigau D (2010). The quality of clinical practice guidelines over the last two decades: a systematic review of guideline appraisal studies. Qual Saf Health Care.

[25] Guidelines International Network. Guidelines International Network Library of Guidelines. 2002.

[26] Institute of Medicine. Clinical Practice guidelines we can trust. 2011.

[27] Schneider Z, Whitehead D. Nursing and Midwifery Research: Methods and appraisal for evidence-based practice 5th edition. LoBiondo-wood G, editor. Australia: Mosby Elsevier; 2014.

[28] Institute JB. The Joanna Briggs Institute Reviewers’ manual. Methodology for JBI scoping reviews. Adelaide, South Australia: University of Adelaide; 2015.

[29] Arksey H, O'Malley L (2005). Scoping studies: towards a methodological framework. Int J Soc Res Methodol.

[30] Aromataris E, Pearson A (2014). The systematic review: an overview. Am J Nurs.

[31] Bowen GA (2009). Document Analysis as a Qualitative Research Method. Qual Res J.

[32] Braun V, Clarke V. Thematic analysis: A practical guide.: Sage Publications.; 2021.

[33] Eccles MP, Grimshaw JM, Shekelle P, Schunemann HJ, Woolf S (2012). Developing clinical practice guidelines: target audiences, identifying topics for guidelines, guideline group composition and functioning and conflicts of interest. Implement Sci.

[34] Garbi M. National Institute for Health and Care Excellence clinical guidelines development principles and processes. Heart. 2021;107(12):949-53. 10.1136/heartjnl-2020-318661 33622678

[35] Hill J, Bullock I, Alderson P (2011). A Summary of the Methods That the National Clinical Guideline Centre Uses to Produce Clinical Guidelines for the National Institute for Health and Clinical Excellence. Ann Intern Med.

[36] Qaseem A, Forland F, Macbeth F, Ollenschlager G, Phillips S, van der Wees P (2012). Guidelines International Network: toward international standards for clinical practice guidelines. Ann Intern Med.

[37] Schünemann H BJ, Guyatt G, Oxman A,. GRADE handbook for grading quality of evidence and strength of recommendations. 2013.

[38] Saunders H (2015). Translating knowledge into best practice care bundles: a pragmatic strategy for EBP implementation via moving postprocedural pain management nursing guidelines into clinical practice. J Clin Nurs.

[39] Lau R, Stevenson F, Ong BN, Dziedzic K, Treweek S, Eldridge S (2016). Achieving change in primary care–causes of the evidence to practice gap: systematic reviews of reviews. Implement Sci.

[40] Wieringa S, Dreesens D, Forland F, Hulshof C, Lukersmith S, Macbeth F (2018). Different knowledge, different styles of reasoning: a challenge for guideline development. BMJ Evid Based Med.

[41] Ansari S. Guidelines for Guidelines: Are They Up to the Task? A Comparative Assessment of Clinical Practice Guideline Development Handbooks. PLoS ONE. 2012. 10.1371/journal.pone.0049864 PMC3506587 23189167

[42] Swiss Centre for International Health. Handbook for Supporting the Development of Health System Guidance: Supporting Informed Judgements for Health System Policies. 2011.

[43] New Zealand Guidelines Group. Ministry of Health 2021 [Available from: https://www.health.govt.nz/about-ministry/ministry-health-websites/new-zealand-guidelines-group.

